# The Oxidative Injury of Extracellular Hemoglobin Is Associated With Reactive Oxygen Species Generation of Grass Carp (*Ctenopharyngodon idella*)

**DOI:** 10.3389/fimmu.2022.843662

**Published:** 2022-02-21

**Authors:** Zhendong Qin, Minxuan Yang, Zhijie Lu, V. Sarath Babu, Yanan Li, Fei Shi, Fanbin Zhan, Chun Liu, Jun Li, Li Lin

**Affiliations:** ^1^ Guangdong Provincial Water Environment and Aquatic Products Security Engineering Technology Research Center, Guangzhou Key Laboratory of Aquatic Animal Diseases and Waterfowl Breeding, College of Animal Sciences and Technology, Zhongkai University of Agriculture and Engineering, Guangzhou, China; ^2^ School of Sciences and Medicine, Lake Superior State University, Sault Ste. Marie, MI, United States

**Keywords:** *Ctenopharyngodon idella*, hemoglobin, oxidative damage, ROS, inflammation, apoptosis

## Abstract

Intravascular hemolysis is a fundamental feature of hemorrhagic venereal infection or tissue and releases the endogenous damage-associated molecular pattern hemoglobin (Hb) into the plasma or tissues, which results in systemic inflammation, vasomotor dysfunction, thrombophilia, and proliferative vasculopathy. However, how the cytotoxic Hb affects the tissues of grass carp remains unclear. Here, we established a hemolysis model in grass carp by injecting phenylhydrazine (PHZ). The data revealed that the PHZ-induced hemolysis increased the content of Hb and activated the antioxidant system in plasma. The histopathology analysis data showed that the PHZ-induced hemolysis increased the accumulation of Hb and iron both in the head and middle kidney. The results of quantitative real-time PCR (qRT-PCR) detection suggested that the hemolysis upregulated the expressions of iron metabolism-related genes. In addition, the immunofluorescence and immunohistochemistry data revealed that the hemolysis caused an obvious deposition of collagen fiber, malondialdehyde (MDA), and 4-hydroxynonenal (4-HNE) accumulation and increased the content of oxidative-related enzymes such as β-galactosidase (β-GAL), lipid peroxide (LPO), and MDA in both the head and middle kidney. Furthermore, the PHZ-induced hemolysis significantly increased the production of reactive oxygen species (ROS), which resulted in apoptosis and modulated the expressions of cytokine-related genes. Taken together, excess of Hb released from hemolysis caused tissue oxidative damage, which may be associated with ROS and inflammation generation.

## Introduction

Hemoglobin (Hb) is an iron-containing metalloprotein whose main biological function is oxygen transport. Under normal circumstances, it is sequestered in the erythrocytes by a highly efficient antioxidant system to prevent vasculature or other tissues from exposure to this pro-oxidative and pro-inflammatory protein (1, 2). However, the extent of hemolysis at the different treatment time points was evaluated using plasma (2–5). Due to the Fenton reaction and pseudoperoxidase (POX) activity [triggered synergistically by microbial proteases and pathogen-associated molecular patterns (PAMPs), such as lipopolysaccharide (LPS) and lipoteichoic acid (LTA)], ferrous Hb-Fe^2+^ is prone to auto-oxidation to ferric Hb-Fe^3+^ (also referred to as methemoglobin) and to ferryl Hb-Fe^4+^, with the oxidized Hb also releasing heme (which is highly redox-reactive and induces oxidative stress) into plasma or tissues (4), leading to the formation of superoxide anion and other reactive derivatives such as hydroxyl radical and hypohalous acid (6–10). Underlying a severe hemolysis, the excess Hb or heme released from RBCs leads to a decreased nitric oxide (NO) bioavailability, which plays an important beneficial role in vascular homeostasis, but otherwise leads to smooth muscle dystonia, vasculopathy, thrombosis, endothelial dysfunction, and platelet aggregation (11–15). Furthermore, excessive high oxidation activity and the pro-inflammatory effects of Hb or heme can overwhelm and impair antioxidants and result in oxidative damage, such as protein oxidation, lipid peroxidation, and nucleic acid oxidation, leading to cellular dysfunction and cell death (6, 16). In human models, studies revealed that intravascular hemolysis and the subsequent release of pro-inflammatory Hb and heme into circulation or tissues are characteristic of several human diseases, including sickle cell disease (SCD), thalassaemias, spherocytosis, paroxysmal nocturnal hemoglobinuria (PNH), autoimmune hemolytic anemia (AIHA), thrombotic microangiopathy (TMA), acute kidney injury (AKI), chronic kidney disease, and atypical hemolytic uremic syndrome (aHUS), among others (11, 17–21).

In recent years, aquaculture has become the fastest-developing field in agriculture, making a significant contribution to global economic stability and food security for human beings (22, 23). Grass carp (*Ctenopharyngodon idellus*) is a worldwide cultured freshwater fish, especially in China. The overall production of grass carp was approximately 5.6 million tons in 2020, which has long ranked first in freshwater aquaculture output in China (24). The continuous expansion of the breeding scale and density of aquaculture, coupled with the deterioration of the breeding environment, all significantly increased the frequency of various bacterial or viral infections, leading to deaths and economic losses. The infection of hemorrhagic pathogens such as *Aeromonas hydrophila* or grass carp reovirus (GCRV) in grass carp causes hemolysis and releases excess Hb to plasma or tissues, which may pose a serious threat to fish health (25). In a previous study, we have demonstrated that the cell-free Hb released from grass carp RBCs could quickly be auto-oxidated to Hb-Fe^3+^ in the presence of oxygen (O_2_) and release superoxide radical (O^2−.^), which could be spontaneously dismutated into H_2_O_2_ and could further oxidize Hb-Fe^3+^ to transient Hb-Fe^4+^. The generated reactive oxygen species (ROS) showed strong bactericidal activity (26). Our further studies also revealed that the grass carp Hb increased the cytotoxicity and secretion of inflammatory cytokines and activated mitogen-activated protein kinases (MAPKs) and nuclear factor kappa B (NF-κB) pathways to generate ROS, resulting in oxidative damage to *C. idellus* kidney (CIK) cells (25, 27). However, the mechanism of hemolysis-induced tissue injury in teleost is still not clear.

Therefore, in this study, we hypothesized that the excess Hb induced by hemolysis *in vivo* may finally alter tissue and vascular homeostasis and thereby promote adverse outcomes in grass carp. To decode this hypothesis, we evaluated a grass carp hemolysis model. Blood, head kidney, and middle kidney were selected to study the effect of Hb. The results of this study will shed new light on blood immunity function in teleost fish.

## Materials and Methods

### Experimental Animals and Treatments

Grass carp (100–250 g) juveniles were obtained from a fish farm located in Guangdong Province, China, and maintained at 28–29°C in a recirculating freshwater system for at least 2 weeks of acclimation to the laboratory condition before carrying out the experiments.

### Hemolysis Model

The hemolysis model was established as described previously, with minor modification (28). Fish were randomly divided into two groups: one group of fish given intraperitoneal injection of PHZ at 40 mg/kg body weight and a control group of fish injected with an equal volume of phosphate-buffered saline (PBS). In time course experiments, fish were sacrificed at 12, 24, 48, and 96 h after PHZ injection.

### Hb Determination

The Hb concentrations in plasma were evaluated as described previously, with slight modifications (10, 26). Briefly, the blood was collected from the caudal vein of each group of fish using heparinized syringes and mixed with 0.7% buffered saline. After centrifugation at 400 × *g* for 10 min at 4°C, the supernatant was obtained and quantified spectrophotometrically at a wavelength of 404 nm using a microplate reader (Molecular Devices, San Jose, CA, USA).

### Total Iron Measurements

The total iron concentrations in the head kidney and middle kidney were measured according to previous reports (11). Briefly, the head kidney and middle kidney (100 mg) were homogenized in double deionized H_2_O at 1:10 (*w*/*v*). The samples were mixed with 500 µl of an acid mixture containing 1 mM HCl and 10% trichloroacetic acid (TCA) and then incubated at 50°C for 1 h. After centrifugation at 15,000 × *g* for 15 min at room temperature (RT), 750 µl of the supernatant was mixed with 250 µl of 20 mg/ml ascorbic acid, followed by 200 µl of ferrozine (0.85% *w*/*v* in hydroxylamine hydrochloride). The samples were developed over 30 min. The absorbance was measured at 560 nm using a microplate reader. A standard curve was generated using an iron solution standard (500 µg/dl).

### Histopathology Analysis

To reveal the effect of hemolysis on tissues, histopathology analysis was performed. The collected samples were fixed in 4% paraformaldehyde (Servicebio, Wuhan, China) for at least 12 h. The tissues were subsequently stained with hematoxylin and eosin (H&E). The iron deposition in tissues was determined with the Perls’ iron stain assay, and the Sirius red staining assay was performed to verify the effect of hemolysis on tissues. Finally, the tissue sections were observed under a microscope (Axiostar Plus, Carl Zeiss, Oberkochen, Germany), and microphotographs were taken using a Canon PowerShot G6, 7.1 megapixels (Canon 219 Inc., Tokyo, Japan).

### Apoptosis Analysis

The apoptosis assay was performed as described previously, with minor modifications (29, 30). TdT-mediated dUTP nick-end labeling (TUNEL) staining was carried out on 5-μm-thick paraffin-embedded sections using a one-step TUNEL apoptosis assay kit (DAPI; Guge Biology, Wuhan, China). The cell nucleus was counterstained with 4′,6-diamidino-2-phenylindole (DAPI) (Servicebio, Wuhan, China) at 25°C for 5 min. Finally, images were captured under a fluorescence microscope (Leica DMI8, Wetzlar, Germany). The caspase-3 activity in the collected tissues was determined by following the manufacturer’s instructions (Solarbio Biology, Beijing, China).

### Western Blot

The Western blotting (WB) process followed a previous procedure, with slight modifications (26, 31). Briefly, following the membrane transfer of proteins by SDS-PAGE, the membranes were blocked in Tris-buffered saline with Tween 20 (TBST) containing 5% skim milk at 37°C for 2 h, followed by incubation with 100 μl of anti-grass carp Hb and heme oxygenase-1 (HO-1) rabbit serum (diluted 1:1,000) at 4°C overnight. After washing three times with TBST, the membranes were incubated with 100 μl of the secondary antibody horseradish peroxidase (HRP)-linked goat anti-rabbit immunoglobulin G (IgG) antibody (diluted 1:10,000) at RT for 1 h. The signals from HRP conjugates were detected using Clarity Western ECL Substrate (Solarbio Biology, Beijing, China). Finally, the membranes were imaged and analyzed using the ChemiDoc™ MP System (Bio-Rad, Hercules, CA, USA).

### Immunofluorescence Assay

The tissue immunofluorescence assay was performed according to a previously described method (32). After dewaxing in Safeclear II, the tissue sections (5 μm) were rehydrated in graded ethanol and then treated in a microwave for 15 min in 10 mM sodium citrate buffer. The tissues were blocked for 2 h with 5% non-fat milk at RT, the grass carp primary antibodies anti-Hb, anti-HO-1, and malondialdehyde (MDA) (1:1,000 in 2% non-fat milk) for 2 h at RT, and then washed three times using PBS with Tween 20 (PBST) buffer. Subsequently, the fluorescein isothiocyanate (FITC)- or Cy3-conjugated anti-rabbit IgG antibody was added (1:10,000 in 2% non-fat milk) and incubated in darkness for 1 h at 37°C. After washing three times, the tissues were stained with DAPI for 5 min at RT. Finally, they were observed under a fluorescence microscope (Olympus BX51, Tokyo, Japan) after rewashing three times with PBST.

### Immunohistochemistry Detection of 4-HNE

After the treatment of tissue sections as above, the sections were blocked in 5% non-fat milk at RT for 2 h and then incubated overnight at 4°C with antibodies against 4-hydroxynonenal (4-HNE) diluted 1:1,000 in PBST containing 2.5% milk. Signal was developed using polymeric peroxidase-conjugated secondary antibody and DAB. All images were acquired under an Olympus BX51 inverted microscope.

### Determination of Related Enzyme Activity

To further explore the effect of hemolysis on tissues, we examined the contents of β-GAL, LPO, MDA, and three kinds of antioxidases (namely, glutathione peroxidase, superoxide dismutase, and catalase) according to the manufacturer’s instructions (Solarbio Biology, Beijing, China).

### Total RNA Extraction and cDNA Synthesis

The method for total RNA extraction was mainly according to our previous reports (27, 32). Briefly, total RNA of the collected tissues was extracted using the TRIzol reagent (Takara, Dalian, China). Approximately 1 μg of total RNA was used to synthesize the first-strand cDNA using HIScript^®^ Q Select RT SuperMix for qPCR (Vazyme, Nanjing, China) following the manufacturer’s instruction and then stored at −20°C.

### Quantitative Real-Time PCR

The expressions of the detected genes in the collected tissues were studied using quantitative real-time PCR (qRT-PCR) in a real-time thermal cycler (Roche, Basel, Switzerland) following the protocol of the SYBR Real-Time PCR Premixture (Vazyme, Nanjing, China). The procedures were as follows: pre-incubation at 95°C for 30 s, followed by 40 cycles of 95°C for 5 s, 55°C for 20 s, and 72°C for 20 s, and finally at 4°C for 5 min. The qRT-PCR for each gene was performed in triplicate and normalized to the values of β-actin. Statistical analysis used the 2^−ΔΔCT^ method. The qRT-PCR for each gene was performed in triplicate and normalized to the values of b-actin, the specific primers are listed in (**Table 1**).

**Table 1:** Primers used in the study.

Primer name	Sequences
*GcTf*-RT-F	AGTTACTATGTCGTGGCGGTTG
*GcTf*-RT-R	ATCCAGCGTTGCGGTTCA
*GcHO-1*-RT-F	ACATGCCTATACACGCTATCTCG
*GcHO-1*-RT-R	GTCACTCCAGGAAATGAGAAGA
*GcTfR1*-RT-F	GATGATGAAATGGAGGCTAACG
*GcTfR1*-RT-R	GGCAATGACAAATCCGCAG
*Gcferrintin*-RT-F	TCCTGTGCTTCGTGCGTGT
*Gcferrintin*-RT-R	ACCTTCAGTCCGTCCTCGTG
*GcHepcidin*-RT-F	TGAAACACCACAGCAGAACGA
*GcHepcidin*-RT-R	CAGCCTTTGTTACGACAGCAGTT
*GcFPN1*-RT-F	ACTCTTCGCTGGCGTCATTG
*GcFPN1*-RT-R	TGGATTTGGTGCGAGGATGA
*GcDMT1*-RT-F	TTCTCATTGACGAACAGCCAG
*GcDMT1*-RT-R	CAAAGGAAAAGAGCCACGGAT
*GcTNFa*-RT-F	GCTGCTGTCTGCTTCACGC
Gc*TNFa*-RT-R	AGCCTGGTCCTGGTTCACTCT
*Gccaspase3*-RT-F	GCATCATCATCAACAACAAA
*Gccaspase3*-RT-R	GACTGAGCATCACACAAACA
*GcIL-1β*-RT-F	GTGTCTGGCCATTTCCAAGAGTA
*GcIL-1β*-RT-R	GGTGTTGAGAGTTTCAGTGACCT
Gc*IL8*-RT-F	GAGTCTTAGAGGTCTGGGTG
Gc*IL8*-RT-R	CAGGTTAAAATATTGTGCAT
*GcTLR4*-RT-F	GAATAATGGGCAGCCGTAAAGTC
*GcTLR4*-RT-R	TCCTCTCTTCCACATCTTCCAGA
*Gc*β-actin-RT-F	ACCCACACCGTGCCCATCTA
*Gc*β-actin-RT-R	CGGACAATTTCTCTTTCGGCTG
*GcIL-4*-RT-F	AATAGGGATCAACGAGAA
*GcIL-4*-RT-R	TGAATGGTTATGTAGGGT
*GcCCL4*-RT-F	TGACAGCATTTGCCATAC
*GcCCL4*-RT-R	GTCCAATACGCATTCCTT
*GcCCL20*-RT-F	CCAGACGCTGTTCAGTTC
*GcCCL20*-RT-R	TTCACCCTCAAAGCCAAT
*GcCCL11*-RT-F	GCGGTGAGGACTCTTTGA
*Gc-CCL11*-RT-R	GTCTGGGACAGTCGCTTC
*GcNrf2*-RT-F	CAATGAGATGATGTCCAAGCACC
*GcNrf2*-RT-R	TTCGCTCTTCTCCTTCTTCAGAC
*GcHap*-RT-F	CTCTCTGTGGCTGTGCTGCT
*GcHap*-RT-R	TCAGTACCCAGCGCTGCGCT

### Statistical Analysis

Statistical analysis was performed using SPSS software (version 17.0). All data were represented as the mean ± standard deviation (SD) of at least three independent experiments. The statistical significance of the data was assessed with one-way analysis of variance (ANOVA), and the figures were drawn in GraphPad Prism 7. Differences were considered significant at *p < 0.05 or **p < 0.01.

## Results

### Hemolysis Caused Hb Plasma Accumulation and Activated Antioxidant System

**Figure 1:**
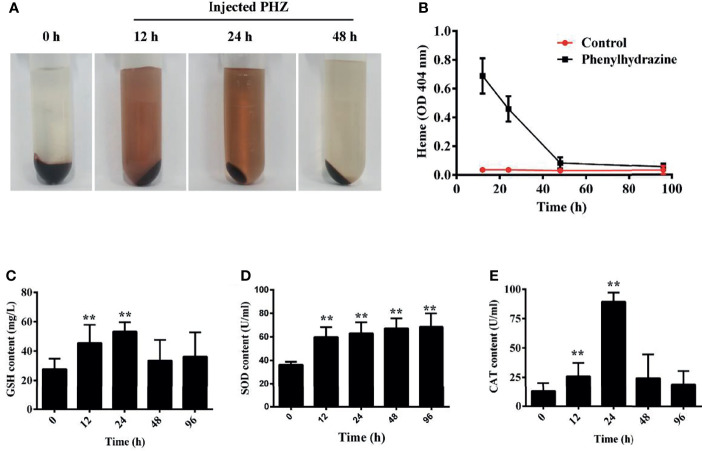
Hemolysis caused hemoglobin (Hb) accumulation in plasma and activated the antioxidant system. **(A)** Plasma was obtained after phenylhydrazine (PHZ) injection for 12, 24, and 48 h **(B)** Hemolysis detection of plasma after injection of PHZ at different time points. **(C–E)** PHZ-induced hemolysis activated three kinds of antioxidases, namely, glutathione peroxidase (GSH) **(C)**, superoxide dismutase (SOD) **(D)**, and catalase (CAT) **(E)**. **p < 0.01 (Student’s *t*-test). Data represent the mean ± SD of three independent experiments.

Here, hemolysis in grass carp was established after intraperitoneal injection of PHZ. The extent of hemolysis at the different treatment time points was evaluated using plasma. The PHZ-treated groups showed obvious Hb accumulation relative to the non-treated groups at 12 and 24 h, and the accumulation of Hb was markedly attenuated after treatment for 48 h (**Figures 1A, B** **)**. To assess whether hemolysis in grass carp affects the antioxidant system, we determined the activity of three antioxidases: glutathione peroxidase (GSH), superoxide dismutase (SOD), and catalase (CAT). After treatment with PHZ, the content of GSH in serum was significantly increased from 12 to 24 h. A significant attenuating effect of GSH was obtained after treatment for 48 and 96 h (**Figure 1C**). Compared to GSH, we observed a significantly increased effect on the SOD contents at all tested time points (**Figure 1D**). For the content of CAT, we found that hemolysis caused the highest effect at 24 h. After treatment for 96 h, the content of CAT showed no significant difference compared to the control groups (**Figure 1E**).

### Hemolysis Increased the Iron Deposition and Injured the Tissues

**Figure 2:**
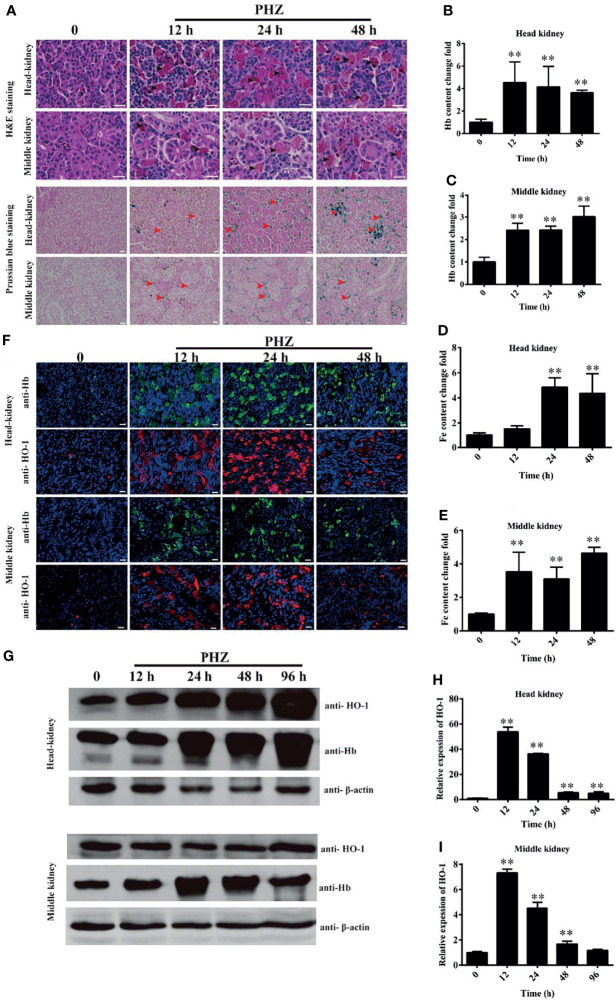
Hemolysis increased the iron deposition and injured the tissues. **(A)** Hematoxylin–eosin (H&E)- and Prussian blue-stained head and middle kidney at 12, 24, and 48 h after phenylhydrazine (PHZ) injection. The *black arrow* denotes several leukocyte-like cells aggregating together to phagocytose the excess hemoglobin, while the *red arrow* represents ion accumulation in the head and middle kidney. *Scale bar*, 20 μm. **(B, C)** Contents of hemoglobin in the head **(B)** and middle **(C)** kidney after PHZ injection at 12, 24, and 48 h **(D, E)** Detection of iron accumulation in the head **(D)** and middle **(E)** kidney after PHZ injection at 12, 24, and 48 h **(F)** Accumulation of hemoglobin and heme oxygenase-1 (HO-1) in the head and middle kidney determined by immunofluorescence assay (IFA) after PHZ injection at 12, 24, and 48 h *Scale bar*, 20 μm. **(G)** Western blotting (WB) was used to analyze the accumulation of hemoglobin and HO-1 in the head and middle kidney after PHZ injection at 12, 24, 48, and 96h **(H, I)** mRNA transcript levels in the head and middle kidney analyzed by qRT-PCR after PHZ injection at 12, 24, 48, and 96 h **p < 0.01 (Student’s *t*-test). Data represent the mean ± SD of three independent experiments.

To evaluate the effect of hemolysis on tissues, multiple staining assays were performed. The results of H&E staining of the head kidney showed an obvious Hb deposition in the PHZ-treated groups, especially in the 24- and 48-h treated groups. Large amounts of Hb were contained in leukocyte-like cells, and the interstitial spaces were enlarged significantly (**Figure 2A**). A similar observation was found in treated middle kidney, where abundant deposits of Hb were mostly engulfed by leukocyte-like cells. Several leukocyte-like cells aggregated together to phagocytose the excess Hb. The hemolysis caused serious damage to the middle kidney, which especially destroyed the integrity of the kidney tubules (**Figure 2A**). To further assess whether the excess Hb caused by hemolysis led to iron accumulation in tissues, we performed the Prussian blue staining assay in the head and middle kidney. The staining results confirmed that the hemolysis resulted in a large amount of ion accumulation both in the head and middle kidney (**Figure 2A**). The accumulation of Hb in the head and middle kidney was also detected by the microplate reader, with the results showing that the PHZ-induced hemolysis in grass carp significantly increased the accumulation of Hb in all tested time points (**Figures 2B, C** **)**. Determination of the deposition of iron using the kit showed that the content of iron in the head kidney was significantly higher at 24 and 48 h compared to the control (**Figure 2D**), and the iron content in the middle kidney was significantly increased in all tested time points (**Figure 2E**). Subsequently, indirect immunofluorescence of Hb and HO-1 was performed to examine whether the iron was derived from the degradation of Hb. Compared to the control groups, the contents of Hb in both the head and middle kidney were significantly higher; however, after treatment for 48 h, the deposition of Hb showed a pronounced attenuating trend (**Figure 2F**). Results of the determination of HO-1 clearly displayed that the hemolysis in grass carp caused a sharp increase in the HO-1 content of both the head and middle kidney, which corresponded to the attenuating trend of Hb (**Figure 2F**). The WB results further verified that Hb increased significantly from 24 to 96 h in the head kidney after treatment with PHZ, which was similar to that determined for the content of HO-1 (**Figure 2G**). In the middle kidney, the WB results showed that the accumulation of Hb rapidly increased at 24 and 48 h after injection of PHZ; however, no significant effect was observed in the hemolysis-induced release of HO-1 in the middle kidney (**Figure 2G**). In addition, we also detected the mRNA expression of HO-1 in the head and middle kidney. As shown in **Figures 2H, I**, the expression level of HO-1 coincided with the results of the WB.

### Hemolysis Activated Iron Metabolism-Related Genes

**Figure 3:**
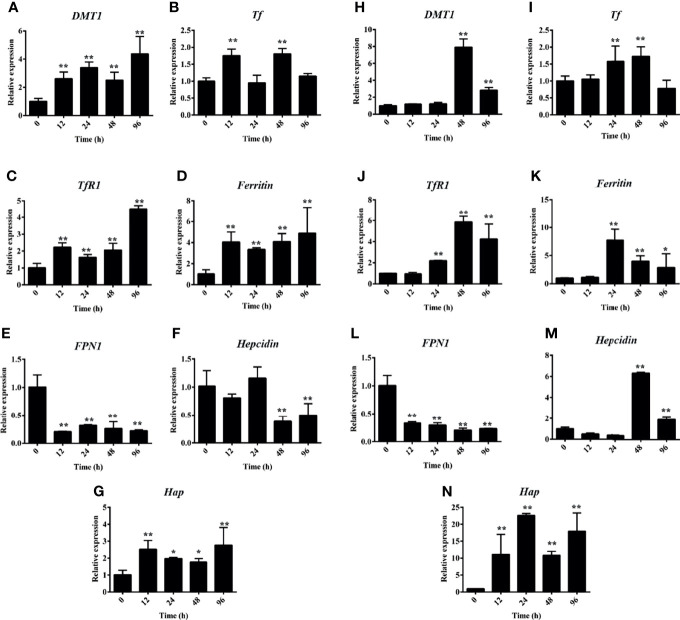
Hemolysis activated the iron metabolism-related genes. **(A–G)** Phenylhydrazine (PHZ)-induced hemolysis activated the expressions of iron metabolism-related genes, including *DMT1* **(A)**, *Tf* **(B)**, *TfR1* **(C)**, *ferritin* **(D)**, *FPN1* **(E)**, *hepcidin* **(F)**, and *Hap* **(G)**, at different time points in the middle kidney. **(H–N)** PHZ-induced hemolysis activated the expressions of iron metabolism-related genes, including *DMT1* **(H)**, *Tf* **(I)**, *TfR1* **(J)**, *ferritin* **(K)**, *FPN1* **(L)**, *hepcidin* **(M)**, and *Hap* **(N)**, at different time points in the middle kidney. *p < 0.05; **p < 0.01 (Student’s *t*-test). Data represent the mean ± SD of three independent experiments.

Accordingly, the PHZ-induced hemolysis caused Hb deposition and iron accumulation in both the head and middle kidney. qRT-PCR was used to further assess whether the PHZ-induced hemolysis modulated the iron metabolism-related genes. The results revealed that the hemolysis significantly increased the expression of divalent metal ion transporter 1 (*DMT1*) at all tested time points in the head kidney and at 48 and 96 in middle kidney (**Figures 3A, H** **)**. Transferrin (*Tf*) was significantly upregulated at 12 and 48 h in the head kidney and at 24 and 48 h in the middle kidney (**Figures 3B, I** **)**. A similar result to *DMT1* was found for the expression of transferrin receptor 1 (*TfR1*), which was significantly upregulated from 12 to 96 h in the head kidney and from 24 to 96 h in the middle kidney (**Figures 3C, J** **)**. The expression level of *ferritin* was significantly increased from 24 to 96 h both in the head and middle kidney (**Figures 3D, K** **)**. Interestingly, the mRNA expression of ferroportin 1 (*FPN1*) was significantly decreased at all tested time points in both head and middle kidney (**Figures 3E, L** **)**. However, the expression of haptoglobin (*Hap*) was significantly increased at all tested time points in both head and middle kidney (**Figures 3G, N** **)**. Lastly, the expression data for *hepcidin* showed a big difference in the head kidney and middle kidney. The expression of *hepcidin* was downregulated at 48 and 96 h in the head kidney, but upregulated in the middle kidney (**Figures 3F, M** **)**.

### Hemolysis Increased the Level of ROS

**Figure 4:**
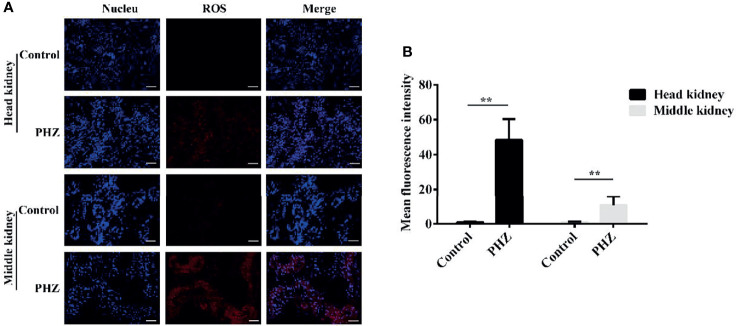
Hemolysis increased the levels of reactive oxygen species (ROS) in the head and middle kidney. **(A)** TUNEL-stained head and middle kidney at 48 h after phenylhydrazine (PHZ)-induced hemolysis. *Scale bar*, 40 μm. **(B)** Mean fluorescence intensity in different groups at 48 h after PHZ-induced hemolysis. **p < 0.01 (Student’s *t*-test). Data represent the mean ± SD of three independent experiments.

**Figure 5:**
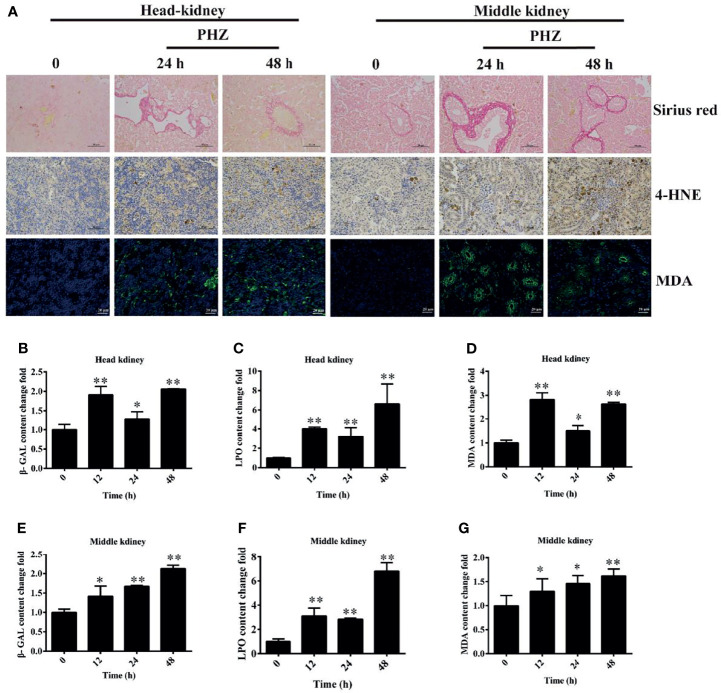
Phenylhydrazine (PHZ)-induced hemolysis caused oxidative damage to tissues. **(A)** Deposition of collagen fiber (shown in *red*) in the head and middle kidney though Sirius red staining assay (*upper* illustration) and the extent of 4-hydroxynonenal (4-HNE) and malondialdehyde (MDA) immunoreactivity, shown in *brown* (*middle* illustration) and *green* (*bottom* illustration), in the tested tissues after PHZ treatment at 24 and 48 h **(B–D)** Contents of three oxidative-related markers—β-galactosidase (β-GAL) **(B)**, lipid peroxide (LPO) **(C)**, and MDA **(D)**—in the head kidney after PHZ injection at 12, 24, and 48 h **(E–G)** Contents of β-GAL **(E)**, LPO **(F)**, and MDA **(G)** in the middle kidney after PHZ injection at 12, 24, and 48 h *p < 0.05; **P < 0.01 (Student’s *t*-test). Data represent the mean ± SD of three independent experiments.

To assess whether hemolysis affects the level of ROS in tissues, we used the dihydroethidium (DHE) assay to detect ROS production after PHZ injection for 48 h. As shown in the data in **Figure 5A**, the hemolysis in grass carp significantly increased the production of ROS in the head kidney compared to the control group. In the middle kidney, the data also revealed that the PHZ-induced hemolysis improved the production of ROS, especially in kidney tubules (**Figure 4A**). To further reveal the effect of hemolysis on the production of ROS, the mean fluorescence intensity of ROS was analyzed. The analysis showed that the PHZ-induced hemolysis significantly increased the mean fluorescence intensity of ROS both in the head and middle kidney (**Figure 4B**).

### Hemolysis Led to Oxidative Damage to Tissues

To directly demonstrate the damage caused by PHZ-induced hemolysis to the head and middle kidney, we firstly performed the Sirius red stain assay. The results showed that hemolysis in grass carp caused an obvious deposition of collagen fiber both in the head and middle kidney (**Figure 5A**). The oxidative markers 4-HNE and MDA were also determined in both head and middle kidney, with the results showing that the PHZ-induced hemolysis significantly increased the content of 4-HNE at 24 and 48 h in the head kidney; similar results were found in the middle kidney (**Figure 5A**). For MDA, the detection results showed that the accumulation of MDA in the head kidney was significantly increased at 24 and 48 h, with similar results revealed for the middle kidney (**Figure 5A**). Furthermore, oxidative-related enzymes were also detected. It was shown that hemolysis significantly increased the content of β-GAL at all tested time points both in the head and middle kidney (**Figures 5B, E** **)**. For the determination of LPO, the results revealed that hemolysis increased its content in both head and middle kidney (**Figures 5C, F** **)**. Similar results were also found in the determination of MDA (**Figures 5D, G** **)**.

### Hemolysis Activated Apoptosis in Tissues

**Figure 6:**
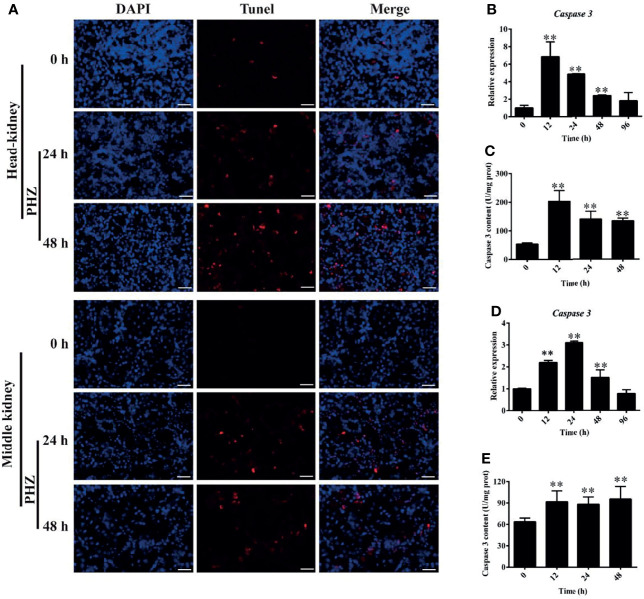
Hemolysis induced apoptosis in the head and middle kidney. **(A)** TUNEL staining showing apoptosis in *red* after phenylhydrazine (PHZ)-induced hemolysis at 24 and 48 h in the head and middle kidney. *Scale bar*, 40 μm. **(B, D)** Expression of caspase 3 in the head **(B)** and middle **(D)** kidney after PHZ-induced hemolysis at 24 and 48 h **(C, E)** Content of caspase 3 in the head **(C)** and middle **(E)** kidney after PHZ-induced hemolysis at 24 and 48 h **p < 0.01 (Student’s *t*-test). Data represent the mean ± SD of three independent experiments.

To further investigate the effect of hemolysis on tissues, we evaluated apoptosis in the head and middle kidney after injection of PHZ for 24 and 48 h. The detection data for the head kidney clearly showed that the hemolysis markedly increased the TUNEL signal after PHZ treatment for 24 and 48 h, which confirmed that it induced apoptosis in the head kidney (**Figure 6A**). A similar result was also found for the middle kidney, in which the PHZ-induced hemolysis significantly increased the apoptosis both at 24 and 48 h compared to the control group (**Figure 6A**). In addition, we also detected the mRNA expression and enzyme activity of caspase 3 in the head and middle kidney after treatment at different time points. The data in **Figure 6B** revealed that the hemolysis markedly increased the expression of caspase 3 from 12 to 48 h in the head kidney. Besides, compared to the control head kidney, the enzyme activity of caspase 3 in the treated groups was significantly increased from 12 to 48 h (**Figure 6C**). In the middle kidney, the PHZ-induced hemolysis significantly increased the mRNA transcript and enzyme activity level of caspase 3 at 12, 24, and 48 h (**Figures 6D, E** **)**.

### Hemolysis Increased Cytokine Expression in Tissues

**Figure 7:**
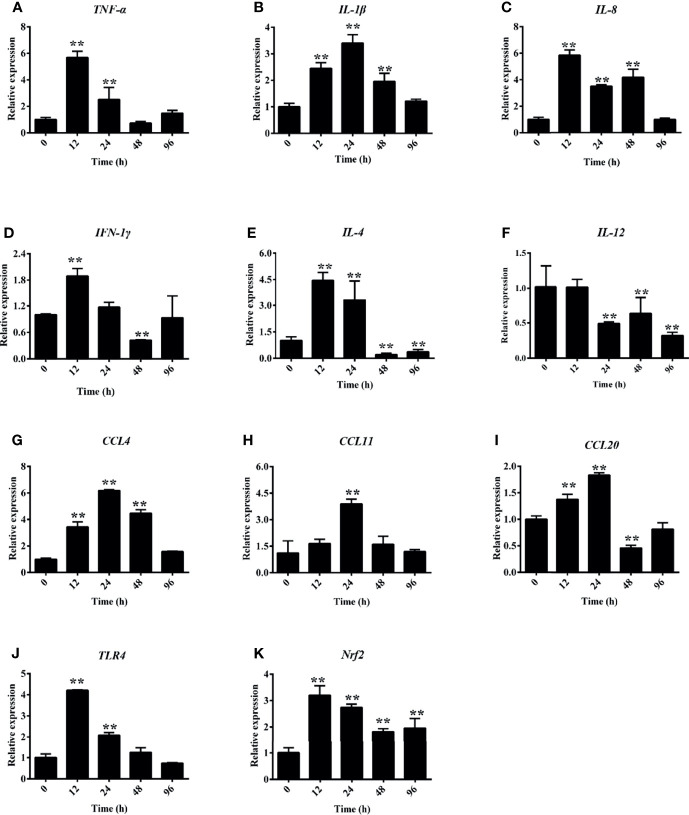
Phenylhydrazine (PHZ)-induced hemolysis caused differential mRNA expression levels of several cytokines measured by qRT-PCR in the head kidney. Cytokines: *TNF-α* **(A)**, *IL-1β* **(B)**, *IL-8* **(C)**, *IFN-1γ* **(D)**, *IL-4* **(E)**, *IL-12* **(F)**. Chemokines: *CCL-4* **(G)**, *CCL-11* **(H)**, *CCL20* **(I)**, *TLR-4* **(J)**, and *Nrf-2* **(K)**. Data are representative of three independent experiments. **p < 0.01 (Student’s *t*-test). Data represent the mean ± SD of three independent experiments.

Under hemorrhagic venereal infection or pathophysiological conditions, hemolysis occurs, which releases large amounts of Hb into the tissues and induces inflammation (6). To evaluate the effect of hemolysis on cytokines, we measured the expressions of several cytokine-regulated genes in the head kidney. **Figure 7A** shows that the hemolysis significantly increased the expression of *TNF-α* at 12 and 24 h in the head kidney. Compared to the control group, the expression levels of *IL-1β* and *IL-8* were both significantly increased after PHZ treatment from 12 to 48 h in the head kidney (**Figures 7B, C** **)**. For *IFN-1γ*, it was found that the mRNA transcript level at 12 h was significantly higher compared to the control group, and no significant effect was observed at other time points (**Figure 7D**). The mRNA transcript levels of *IL-4* at 12 and 24 h were significantly increased (**Figure 7E**); however, the expression of *IL-12* was more noticeably attenuated from 24 to 96 h (**Figure 7F**). In addition, we also detected the expressions of three chemokine genes, with the detection data revealing that PHZ-induced hemolysis activated their expressions to varying degrees. The detection data for *CCL4* revealed that its mRNA transcript level reached the highest point after 24-h treatment, followed by that at 48 and 24 h (**Figure 7G**). The expression of *CCL11* was significantly increased at 24 h compared to the control group (**Figure 7H**). For *CCL20*, its mRNA transcript level was significantly upregulated at 12 and 24 h; however, its expression at 48 h was downregulated (**Figure 7I**). Finally, we also detected the expressions of *TLR4* and *Nrf2*. Their data revealed that the hemolysis significantly upregulated the mRNA transcript level of *TLR4* at 12 and 24 h (**Figure 7J**). The hemolysis significantly increased the expression of *Nrf2* at all tested time points (**Figure 7K**).

## Discussion

Under normal physiological conditions, Hb is encapsulated in red blood cells, which is widely acknowledged. Intravascular hemolysis can occur in a pathological state, such as during tissue injury, stored RBC transfusions, or hemolytic microbe infection (2, 33). Exposure to high levels of free Hb or heme occurs in several disease states (such as sickle cell disease and kidney injury-associated diseases), which is widely studied in mammal models. Similarly, in the grass carp farming process, infection of *A. hydrophila*, GCRV, or other hemorrhagic pathogens usually leads to severe hemolysis and releases high levels of Hb into plasma or tissues (34, 35). In this study, we established a hemolysis model in grass carp to reveal the effect of Hb on tissues. Firstly, we explored the Hb accumulation in plasma after injecting PHZ at different time points. The data showed that Hb was deposited rapidly within a short period of hemolysis, which was similar to transfusion with old blood leading to intravascular hemolysis in guinea pigs (36). However, the accumulation of Hb was significantly attenuated after PHZ injection for 48 h, which indicated that the grass carp possessed a Hb scavenging system. Reports in mammals expounded that Hap and hemopexin (Hpx) play the role of primary scavenger proteins for the clearance and detoxification of extracellular Hb and hemin (17, 37); however, the scavenger system in teleost is still not well known. In our study, the PHZ-induced hemolysis also increased the antioxidant system; however, the enzyme activity patterns of the three antioxidases were obviously different. The exact mechanism of the different antioxidant enzymes in hemolysis still needs to be further deciphered in grass carp.

Due to the Fenton reaction, cell-free Hb contains a high oxidative activity. The presence of cell-free Hb and heme in circulation or tissues induces a cascade of oxidative reactions, resulting in systemic inflammation and widespread tissue damage (6, 17). In a guinea pig model, at 24 h after transfusion of old blood, HE staining demonstrated an extensive luminal to medial coagulative necrosis in distinct vascular regions of proximal and distal tubular dilation, necrosis in the kidney, and swollen tubules with irregular shapes, orange-colored casts, and irregular distribution of nuclei in dilated proximal and distal tubules. Perls’ iron staining showed obvious iron deposits appearing at the corresponding locations (36). In this study, we also tested the injury of Hb induced by hemolysis through H&E and Perls’ iron staining assays. The data revealed that hemolysis caused serious damage to the middle kidney, especially destroying the integrity of the kidney tubules and with large amounts of iron accumulation both in head and middle kidney. Under several pathological conditions associated with extensive hemolysis, the protective effect of a scavenger system can be rapidly overwhelmed. Cell-free Hb accumulates in plasma and is oxidized, releasing its heme groups, which can catalyze the production of free radicals through Fenton chemistry, resulting in oxidative damage (38, 39). Under oxidative stress, the host cells can avoid the pro-oxidant effects of free heme mainly through the rapid induction of the HO-1 isoenzyme, which catabolizes free heme into equimolar amounts of Fe^2+^, carbon monoxide (CO), and biliverdin to prevent the cells from inducing programmed cell death in response to pro-inflammatory agonists in a variety of mechanisms (39, 40). Our study revealed that the PHZ-induced hemolysis significantly increased the expression of *HO-1* both in head and middle kidney, which indicated that the *HO-1* in grass carp kidney plays a vital role affording protection against cell-free heme, thus exerting salutary effects against oxidative damage. This result is consistent with our previous study *in vitro* (41). In this study, we have demonstrated that the PHZ-induced hemolysis resulted in the accumulation of excess intracellular iron and tissue damage, destroying systemic iron homeostasis. In a mouse model, excess Hb destroyed the iron homeostasis and activated the expression of iron metabolism-related genes, similar to the results found in this study (42, 43).

Previous studies revealed that the oxidized Hb releases heme, and the generation of free radicals by the Fenton reaction has been considered the major form of ROS generation by heme and an important mechanism of heme-induced cytotoxicity (40, 44). Our previous studies also revealed that Hb activated the MAPK and NF-κB pathways to generate ROS, resulting in oxidative damage to CIK cells (25). The results from this study also revealed that the PHZ-induced hemolysis obviously increased the production of ROS in both head and middle kidney. Subsequently, we also examined the oxidative stress of hemolysis in the head and middle kidney. The results showed an obvious deposition of collagen fiber in the tested tissues. 4-HNE and MDA were also significantly accumulated, as well as the oxidative-related enzymes β-GAL and LPO, which were significantly increased. These results suggested that the PHZ-induced hemolysis caused oxidative damage to both head and middle kidney. Prolonged exposure to high levels of ROS usually leads to apoptosis, and thereby is widely acknowledged. In murine polymicrobial sepsis, cell-free Hb increased lung apoptosis (45). In a human podocyte model, during intravascular hemolysis, podocytes take up Hb, promoting oxidative stress, podocyte dysfunction, and apoptosis (18). In our previous *in vitro* study, high levels of Hb caused apoptosis in CIK cells (41). Here, we also examined the effect of hemolysis on apoptosis in both head and middle kidney through the TUNEL assay. The data revealed that the PHZ-induced hemolysis increased apoptosis compared to the control group, which indicated that the hemolysis in teleost also caused oxidative damage and resulted in apoptosis, similar to the reports in mammals. Cell-free Hb or heme plays the role of a pro-inflammatory molecule, which has been widely reported in several cells. According to previous studies, Hb or heme could induce inflammatory reactions *via* a Toll-like receptor 4 (TLR4)-dependent mechanism in macrophages (46), NF-κB activation in endothelial cells (47, 48), and leucine-rich repeat-containing protein 3 (NLRP3) in both macrophages and endothelial cells (48, 49). In CIK cells, we also found that the incubation of heme protein increased the secretion of inflammatory cytokines such as *IL-6*, *CCL1*, *TNF-α*, and *IL-1β* (25). In this study, the PHZ-induced hemolysis significantly increased the expressions of the cytokine-related genes in the head kidney; however, the expression pattern of these cytokine-related genes showed some differences. The mechanism of the regulation of Hb in cytokines still needs further elucidation.

In sum, we aimed to evaluate the effect of Hb released from PHZ-induced hemolysis on grass carp head and middle kidney. Our data demonstrated that the PHZ-induced hemolysis increased Hb and iron accumulation, activated the expressions of iron metabolism-related genes, increased the production of ROS in both head and middle kidney, and resulted in oxidative damage and inflammation generation.

## Data Availability Statement

The original contributions presented in the study are included in the article/supplementary material. Further inquiries can be directed to the corresponding authors.

## Ethics Statement

The animal study was reviewed and approved by the Animal Ethics Committee of Zhongkai University of Agriculture and Engineering.

## Author Contributions

ZQ performed experiments, analyzed the data, and wrote the manuscript. MY, ZL, VSB, YL, FS, FZ, and CL performed the experiments. JL and LL conceived ideas, analyzed the data, oversaw the research, and wrote the manuscript. All authors contributed to the article and approved the submitted version.

## Funding

This work was jointly supported by the National Natural Science Foundation of China (31902409, 31872606, 31572657, and U1701233); Special Funds for Economic Development of Marine Economy of Guangdong Province (2019A1); Foundation of Guangdong Provincial Marine and Fisheries Bureau (GDME-2018C006 and D21822202); and the Foundation of China–ASEAN Maritime Cooperation (CAMC-2018F). JL was supported by the Pearl River Scholarship from Guangdong Province.

## Conflict of Interest

The authors declare that the research was conducted in the absence of any commercial or financial relationships that could be construed as a potential conflict of interest.

## Publisher’s Note

All claims expressed in this article are solely those of the authors and do not necessarily represent those of their affiliated organizations, or those of the publisher, the editors and the reviewers. Any product that may be evaluated in this article, or claim that may be made by its manufacturer, is not guaranteed or endorsed by the publisher.
